# Epigenetics in myeloproliferative neoplasms

**DOI:** 10.3389/fonc.2023.1206965

**Published:** 2023-07-13

**Authors:** Graeme Greenfield, Mary Frances McMullin

**Affiliations:** Department of Haematology, Queen’s University, Belfast, United Kingdom

**Keywords:** epigenetics, myeloproliferative neoplasms, DNA methylation, JAK2, NFE2, histone modification

## Abstract

The myeloproliferative neoplasms (MPNs) are a group of acquired clonal disorders where mutations drive proliferative disease resulting in increased blood counts and in some cases end-stage myelofibrosis. Epigenetic changes are the reversible modifications to DNA- and RNA-associated proteins that impact gene activity without changing the DNA sequence. This review summarizes mechanisms of epigenetic changes and the nucleosome. The drivers and epigenetic regulators in MPNs are outlined. In MPNs, distinct patterns of epigenetic dysregulation have been seen in chronic and in advanced phases. Methylation age and histone modification are altered in MPNs and by further treatment. The alterations found in methylation age in MPNs and with treatment are discussed, and the changes in histone modification with Janus kinase (JAK) inhibition are evaluated. Currently available therapeutic areas where the epigenome can be altered are outlined. Thus, we review the current knowledge and understanding of epigenetics in MPN and consider further management options. Understanding the epigenome and its alteration in MPNs and epigenetic changes associated with the progression of disease will lead to advances in therapeutic options.

## Epigenetics

Genomic instability is fundamental to the development of malignancy. Acquired mutations may drive the process, but dysregulation of the normal epigenetic mechanism is frequently observed across nearly all forms of hematological malignancy and solid tumors (1). Epigenetic changes include reversible modifications to chromatin structure, histone modifications, and DNA methylation, which dictates the way genes can be expressed or silenced. Maintaining a particular gene transcription profile is critical to the normal function of the cell. Thus, in contrast to changes in the genetic code, with epigenetic dysregulation, there are no DNA sequence changes. Alterations in chromatin structure or modification of histones, DNA methylation, or changes to RNA can result in downstream gene expression changes that may influence the initiation, maintenance, or progression of a malignant cell (2).

**Figure 1:**
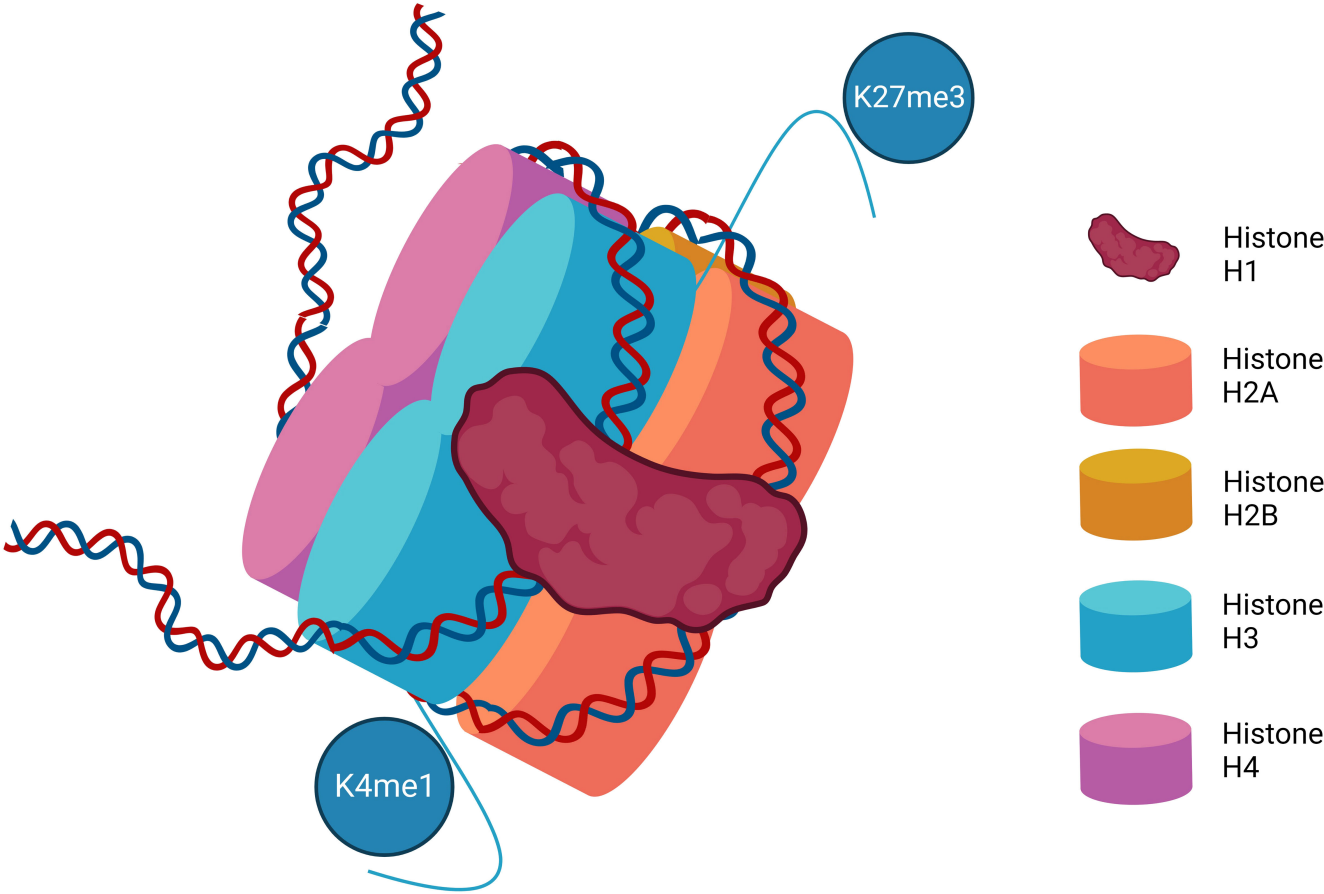
The structure of the nucleosome. Represented are the eight histone proteins that form the core of the histone along with histone H1. Various methylation marks are illustrated on the N terminal amino acid chains depicting examples of the more common and best characterized posttranslational histone modifications that impact DNA transcription. K4me1–Lysine 4 mono-methylation is considered mark of active and primed enhancers. K27me3–Lysine 27 trimethylation is a mark of gene repression.

In cells, DNA is packed into chromatin in the nucleosome. The DNA is coiled round a histone protein core of eight histone proteins (two each of H2A, H2B, H3, and H4). Histone H1 further stabilizes the structure. This is not a static structure, as changes in the condensed nature of the chromatin signal for changes in gene transcription (3) (**Figure 1**). Posttranslational modifications of histone proteins are one of the most studied and best understood mechanisms of epigenetic control. Histone modification predominantly occurs on histone N terminal residues with specific changes driving transcriptional activation or repression. These modifications primarily include acetylation, methylation, and phosphorylation. Other histone posttranslational modifications including butyrylation and sumoylation (4) are increasingly recognized, but their relevance in MPN is unclear and they will not be discussed in this review.

Proteins involved in the modification of histones are broadly classed as writers, erasers, and readers. Writers are responsible for the deposition of a particular chemical modification, while erasers remove these modifications. Readers recognize modifications and recruit additional proteins to enhance or repress transcription. Histone acetylation is reversibly regulated by histone acetyltransferases (writers) and histone deacetylases (HDACs) (erasers). Particularly on histones H3 and H4, acetylation occurs at lysine (K), while HDACs remove acetyl residues from histone tails (5).

Histone acetyltransferases and HDACs are recruited to target genes with specific factors and regulate gene expression. HDACs can be divided into three classes. Class I HDACs (1, 2, 3, and 8) are located in the nucleus, Class II HDACs (4–9) are located in the nucleus and cytoplasm, and Class III HDACs are distinct NAD-dependent enzymes (6). Class I and II HDACs are inhibited by suberoylanilide hydroxamic acid (SAHA) and other HDAC inhibitors inducing growth arrest, differentiation, apoptosis, and inhibition of tumor growth in cancers including hematological malignancy (7).

Methylation of histones takes place on lysine, arginine, and histidine residues. However, in addition, lysine may be mono-(me), di-(me2), or tri-(me3) methylated. S-adenosyl methionine (SAM) donates the methyl group by the action of histone methyltransferase (HMT) (10). Enhancer of Zeste 2 (EZH2) is the enzymatic component of the Polycomb repressive complex 2 (PRC2) and is a prime example of an HMT, which is frequently dysregulated in cancer and may act as a therapeutic target (8). Histone demethylase enzymes including lysine-specific demethylase 1 (LSD1) act as erasers in this context to remove methylation marks (9). Histone methylation marks frequently studied are histone H3 lysine 4 (H3K4) associated with transcription activation in its trimethylated state and histone H3 lysine 9 (H3K9) associated with transcriptional repression. The balance between acetylation and methylation histone H3 lysine 27 (H3K27) is important for determining gene expression. Trimethylation of H3K27 (H3K27me3) is associated with transcriptional repression, while acetylation (H3K27ac) is a mark of active gene transcription (11, 12).

**Figure 2:**
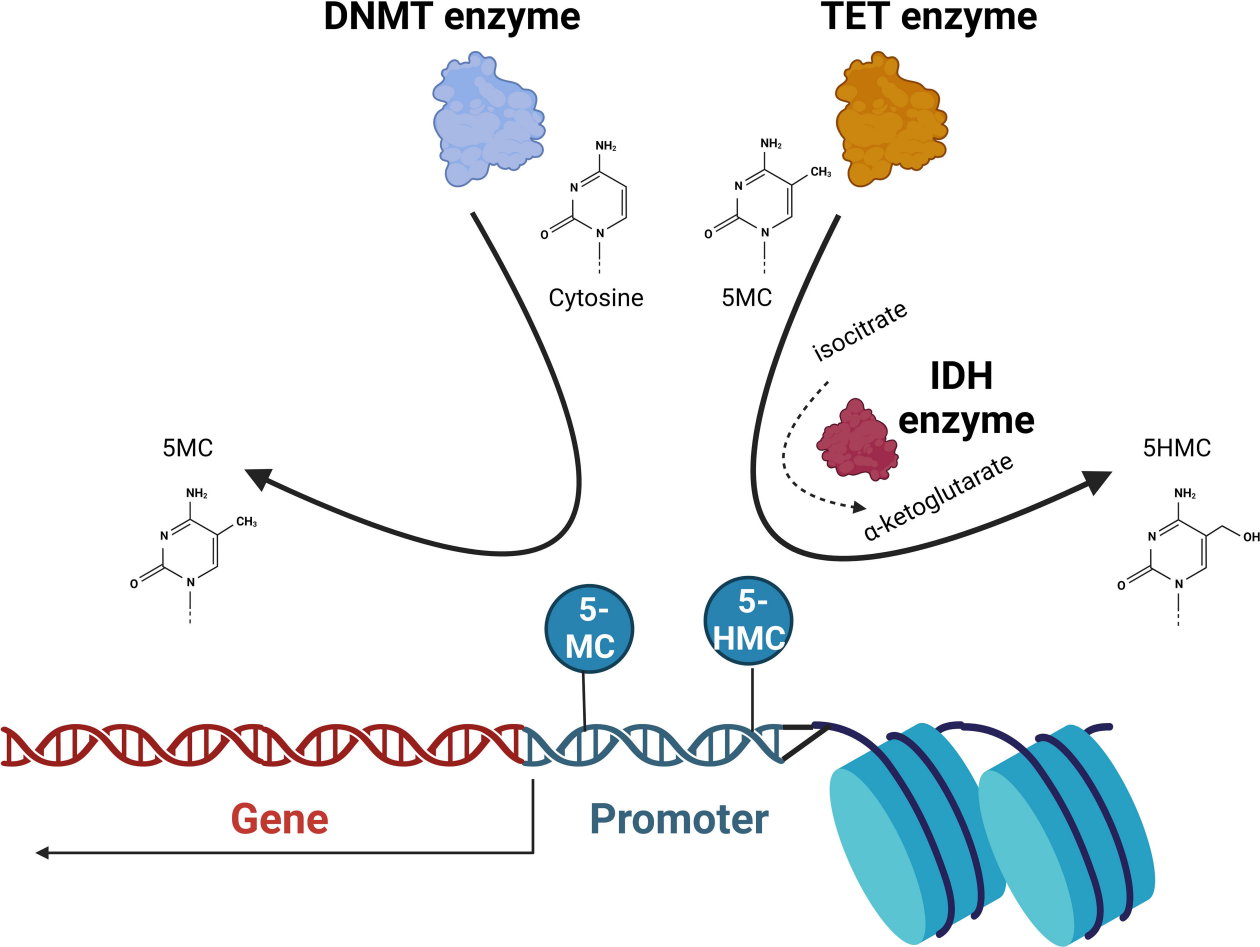
DNA methylation. The DNA methyltransferase (DNMT) enzymes are responsible for the conversion of cytosine to 5-methylcytosine (5-MC) that at promoter regions acts to repress transcription. Ten-eleven translocation (TET) enzymes convert 5-MC to 5-hydroxymethylcytosine (5-HMC) that acts to de-repress transcription. Isocitrate dehydrogenase (IDH) proteins convert isocitrate to α-ketoglutarate that the TET2 is dependent on. The balance of unmethylated, methylated, and 5-hydroxy methylated cytosines present in promoter regions will ultimately determine the transcriptional activation of the gene.

DNA methylation is a well-described epigenetic mechanism where the highly conserved methylation of chromatin components is involved in the regulation of gene expression, DNA repair and replication. Cytosines are methylated by the addition of a methyl group to the pyrimidine ring by the action of DNA methyltransferase (DNMT) enzymes. This occurs mainly at CpG islands (regions where a cytosine nucleotide is followed by a guanine). CpG islands are found across the genome and are present in the promoter regions of the majority of genes in humans. The 5-methylcytosine (5-mc) formed represses gene transcription. SAM donates a methyl group (CH3) and is reduced to S-adenosyl homocysteine (SAH). The ten-eleven translocation (TET) proteins catalyze the conversion of 5-mc to 5-hydroxymethylcytosine (5-hmc), which is an initial step in demethylating DNA. The isocitrate dehydrogenase (IDH) enzymes catalyze the conversion of isocitrate to a-ketoglutarate, a reaction that is required for TET enzyme function. Ultimately, unmethylated DNA with an open chromatin structure is involved in active transcription, whereas with methylated DNA and a closed chromatin structure, transcription is impeded (13) (**Figure 2**).

RNA changes in regulation can lead to cell cycle arrest and apoptosis. Mutations in the genes controlling RNA regulation, *SF3B1 (Spicing factor 3b subunit2)*, *SFSF2 (Serine and arginine rich splicing factor 2)*, and *IKZF1* (*IKAROS family finger 1*) have been described and may affect these processes in myeloid cancers including MPN (14, 15).

## Myeloproliferative neoplasms (MPNs)

The typical Philadelphia-negative myeloproliferative neoplasms (MPN)s are polycythemia vera (PV), essential thrombocythemia (ET), and myelofibrosis (MF). This group of disorders is characterized by acquired clones that drive excess mature myeloid cell production and, in the case of MF, the secondary phenomenon of excess fibrosis. In each of these conditions, driver mutations have been detected that suggest that the acquired clone is accounting for the excess cell production (16).

Cytokines such as erythropoietin (EPO) regulate normal hematopoiesis by stimulating receptors on the cell surface and activating the Janus kinase (JAK) protein. This protein then autophosphorylates and binds to signal transducer and activator of transcription (STAT) protein. STAT proteins then phosphorylate, dimerize, and translocate to the nucleus where they bind at promoter sequences in the genome. This promotes gene transcription that ultimately affects cell functions including cell growth, differentiation, and apoptosis (16). There is then a mechanism to turn off this process *via* the negative regulators including the phosphatase Src homology1 domain (SHP1). In PV, the majority of cases have been found to have a single mutation in the *JAK*2 gene *JAK*2V617F (17), and a small minority have alternative mutations in exon 12 of *JAK*2 *(* 18). These gene mutations lead to constitutively activated proteins that result in upregulation of the JAK-STAT pathway. Activation of alternative intracellular signaling cascades including MAPK and PI3K has also been observed as a result of the JAK2 mutation.

In 50% of those with ET, the *JAK*2V617F mutations can be determined. In cases of ET that do not have this driver mutation, approximately 15% have been found to have mutations in the myeloproliferative leukemia virus oncogene (*MPL*) (19). This gene encodes the thrombopoietin (TPO) receptor protein. The activated receptor stimulates signaling *via* the JAK-STAT pathway controlling the production of blood cells. Again, mutated *MPL* produces a constitutively activated protein and increased cell production. *JAK*2 and *MPL* mutations have been found also in MF.

In some of the ET and MF patients negative for *JAK*2 and *MPL* changes, mutations in the gene calreticulin (*CALR*) have been identified. In approximately 70%–84% of MPN patients with nonmutated *JAK*2, somatic *CALR* mutations have been described (20). The CALR protein is present in the endoplasmic reticulum where it ensures proper glycoprotein folding, contributes to calcium homeostasis, and plays an important role in the unfolded protein response (UPR). Mutant CALR proteins interact with the TPO receptor (MPL) leading to dimerization and activation of JAK2 and activation of the downstream pathway (21, 22). There remain a few ET and MF cases in which no driver mutations have yet been identified so-called “triple-negative” cases.

JAK2 signaling is involved in numerous biological processes in many cell types. This includes cell cycle progression, mitotic recombination, apoptosis, genetic instability, and alteration of heterochromatin (22–24). It has been shown that a significant fraction of JAK2 is present within the nucleus and that it directly phosphorylates tyrosine 41 on histone H3 (H3Y41). In the nucleus, JAK2 mediates the phosphorylation of H3 and displaces heterochromatin protein 1 alpha (HP1α) from a novel binding site surrounding H3Y41. While the displacement of HP1α is tightly regulated in normal cells, with constitutively activated mutated JAK2, unregulated displacement of chromatin-bound HP1α may override its potential tumor-suppressive functions (25).

In addition, the type II arginine methyltransferase, protein arginine methyltransferase 5 (PRMT5) that mediates dimethylation of arginine residues within histones H2A, H3, and H4, contributes to the JAK2 mutant-induced MPN phenotype. JAK2 mutants bind PRMT5 more strongly than wild-type JAK2, phosphorylating PRMT5 and impairing its ability to methylate its histone substrates. This represents a gain of function, allowing the regulation of chromatin modifications (26).

## Mutations in epigenetic regulators

In MPNs, besides the above-described driver mutations, there are a number of genes involved in epigenetic regulation and messenger RNA (mRNA) splicing that are commonly mutated in MPNs. These mutations occur across different myeloid malignancies being not specific to MPNs. Of those, the three most commonly identified pathogenic mutations in MPN patients involve the epigenetic regulators *TET2*, *ASXL1*, and *DNMT3A*, all present at frequencies above 5% in an unselected MPN population (1). *EZH2* mutations are less common in MPN, occurring in approximately 2% of patients overall, but appear particularly important in determining disease progression (27).

TET2 is a member of the α-ketoglutarate-dependent enzyme family that catalyzes the conversion of 5-methylcytosine of DNA to 5-hydromethylcytosine and then induces DNA methylation. *TET*2 mutations have been reported throughout the gene and such loss-of-function mutations result in DNA hypermethylation. *TET*2 mutations have been reported in 12% of a series of MPN patients (28). The prognostic impact of *TET2* mutations is less clear. The sequence of mutation acquisition appears to be important. Hematopoietic stem cells that have a *TET2* mutation occurring first show enhanced self-renewal but lack the proliferative drive to produce excess mature cells. A *JAK2* V617F second hit is then required to produce the MPN phenotype. The opposite is seen in *JAK2* V617F first, *TET2* second cells. Individuals with *TET2* first tend toward an ET phenotype, whereas those *JAK2* V617F first cells tend toward a PV phenotype (29). DNA methyltransferase 3A (*DNMT3A*) encodes for the protein that is responsible for the *de novo* methylation of CpG dinucleotides. Loss-of-function mutations are found in various MPN types (30). It is thought that the epigenetic deregulation resulting from these mutations leads to upregulation of the hemopoietic stem cell (HSC) fingerprint genes (31).

Genes involved in histone methylation are also mutated in MPNs. PcG EZH2 is the catalytic component of the PRC2 and is involved in the trimethylation of histone H3 lysine 27 resulting in transcriptional repression. In MPNs, *EZH*2 mutations tend to be loss-of-function mutations (32) that result in derepression of numbers of oncogenes and are associated with increased HSC self-renewal (33). This is in contrast to the gain-of-function mutations more commonly observed in lymphoid malignancy. *EZH2* mutations are more commonly observed in primary MF. In *JAK2* V617F murine models of MPN, *EZH2* mutations induce a myelofibrosis-like phenotype. This is associated with the loss of H3K27me3 and an epigenetic switch to H3K27ac (34). In this mouse model, the addition of an *EZH2* mutation was observed to drive a bias toward megakaryopoiesis. This highlights the potential for epigenetic changes to control the differentiation capacity of the mutant stem and progenitor cells and ultimately change the resulting disease phenotype.

Additional sex combs like 1 (ASXL1) is an enhancer of the polycomb complexes and regulates both PRC1 and PRC2 (35). *ASXL*1 mutations are associated with the reduction of H3K27 methylation and *HOXA* upregulation, which are both linked to impaired recruitment of the PRC2 complex in particular of EZH2 (36) and occur in MF (37). In a mutant ASXL1-induced model of myelodysplasia (MDS), depression of *HOXA9* was also observed to be related to a reduction in H3K27 methylation (38). Isocitrate dehydrogenase 1 and 2 (*IDH*1 and *IDH*2) catalyze the conversion of isocitrate to α-ketoglutarate. Mutations in a small percentage of MPN cases have been described (39). These mutations result in the production of 2-hydroxyglutarate that inhibits Jumonji-C domain histone methylases leading to histone hypermethylation. While the presumed effects of IDH1/2 mutations also include the hypermethylation of DNA promoter sequences and gene repression, the exact consequences for individual promoters and gene transcription are less clear (40).

Ikaros, a Kruppel-like zinc finger transcription factor, interacts with the histone deacetylase repressor complexes and resulting in a repressive effect on genes involved in myelopoiesis. The *IKZF1* influences maturation and differentiation. Deletions in *IKZF*1 have been found in 21% of MPN patients in blast phase but only in 0.2% of chronic phase patients (41), making it highly possible that *IKZF*1 has a role in leukemic transformation in MPN.

Therefore, a number of mutations found in MPNs affect the regulation of DNA and histone methylation in the HSC compartment. The importance of these mutations is also reflected in the prognostic relevance of each. *ASXL1, EZH2, IDH1/2*, and *TET2* have all been implicated in the progression to fibrotic or leukemic transformation and reduced survival (27).

## Nuclear factor erythroid 2 (NFE2)

Nuclear factor erythroid 2 (NFE2) is a transcription factor that is overexpressed in the majority of MPN patients (42, 43). NFE2 is functioning in chromatin remodeling and gene transcription. *In vivo* mouse models with elevated NFE2 levels have an MPN phenotype (44). In MPN patients, insertions and deletions of NFE2 have been described in approximately 2% of patients. *NFE2* mutations were identified as a predictive variable in determining the risk of fibrotic transformation in a large cohort of individuals with MPNs with chronic stage disease (1). These mutations result in truncated NFE2 proteins that enhance wild-type NRE2 function. NFE2 mutant cells have a proliferative advantage (45). The target genes of NFE2 include the histone demethylase, Jumonji domain containing C (*JMJD1C*), resulting in elevation of JMJD1C levels in PV and MF. Levels of the histone marks H3K9m1 and H3K9me2 are decreased. JMJD1C and NFE2 participate in an autoregulatory loop. NFE2 is independently regulated through the JAK2 epigenetic pathway by phosphorylation of H3Y41 (46). The exploration of these pathways may lead to the discovery of durable targets.

## Epigenetic studies of MPN

In general, DNA methylation has been studied on a gene-by-gene basis as well as using comprehensive methylation profiling aiming at a genome-wide characterization of epigenetic markers in MPNs. In a cohort study of PV, ET, and MF patients, global DNA methylation profiling was investigated, including some patients who had transformed to acute myeloid leukemia (AML) (71 in chronic phase and 13 transformed). MPN samples showed an aberrant methylation pattern compared to control samples, but patterns were similar in all three MPN types. The gene network involved in the NF-κB pathway showed enrichment of differentially methylated regions. The transformed AML cases had an increased number of differentially methylated regions compared to the chronic cases. Therefore, altered DNA methylation may have a role in the pathogenesis of leukemic transformation in MPNs (47). A previous study of 35 MPN patients found homogeneous methylation patterns in MPN subtypes and controls (48). Another study of 29 MPN chronic phase patients showed that PV and ET were characterized by aberrant promoter hypermethylation, whereas MF was epigenetically distinct with aberrant hypo and hypermethylation. Cases with *ASXL1* and *TET2* mutations had distinct epigenetic signatures revealing methylomic signatures for these mutations (49).

## Methylation age

DNA methylation (DNAm) is a well-defined epigenetic mechanism of transcription modification. It is affected by aging, lifestyle, diet, and disease. It is possible to calculate the methylation age of a range of tissues (3). Vorinostat (MK-0683) (suberoylanilide hydroxamic acid) is a pan-histone deactylase inhibitor (HDACi) that has been shown to induce tumor cells to undergo growth arrest, differentiation, and apoptosis (50–52). This agent has been trialed in patients with PV and ET with some responses including decreased leukocyte and platelet counts and modest reductions in *JAK*2V617F allele burdens. Treatment discontinuation rates were high due to toxicity (53).

Methylation age (MA) may be a more accurate reflection of disease than chronological age (CA). Using the aging signature of Weidner et al. (54) to generate individual MA, it was explored whether DNAm is altered in MPN and whether the HDACi vorinostat altered the MA in PV and ET patients who were treated in the trial.

Having verified the aging signature, an older MA was observed in patients with a higher *JAK*2V617F allelic burden and in those with longer duration of disease. PV had an older MA than predicted, whereas it was younger than predicted in ET, perhaps related to the mutant allele burden. Treatment with vorinostat resulted in a younger MA in PV patients and an older MA in ET patients resulting overall in a trend to normal CA. Comparing MA and response, nonresponse was associated with a younger than predicted MA in ET patients and an older than predicted MA in PV patients. There would appear to be a link between MA and *JAK*2 mutant allele burden implying that the allelic burden is not only influencing the clinical phenotype, and disease evolution, but also the overall methylation landscape of the MPN cells (55).

## Histone modification with JAK inhibition

Therapeutic options for MPNs are limited, and the main drug licensed to treat MF is the JAK1/2 inhibitor ruxolitinib. This drug is effective in reducing symptom burden, cell counts, and spleen volume in PV and MF (56). Disease-modifying effects are modest with some reductions in *JAK*2 allele burden (57), but transformation to myelofibrosis and acute leukemia is not altered by ruxolitinib (58). Epigenetic dysregulation may have a role in MPN, and the effect of JAK inhibitor therapy on the epigenetic landscape is of interest in understanding the benefits of treatment. Histone modification was therefore explored in MPN cell lines and in patient samples from the randomized controlled trial MAJIC, which evaluated the efficacy of ruxolitinib versus best available therapy in a second-line setting in PV and ET (59).

After establishing a dose of ruxolitinib in MPN cell lines that showed inhibition of phosphorylation of STAT3 and STAT5 and therefore being sufficient to exert molecular responses in cell lines but not inducing cell death, histone modification was investigated using a 100-nM concentration of ruxolitinib. An increase in methylation marks was seen in the MPN cell lines SET-2, UKE-1, and HEL, showing that ruxolitinib treatment of cell lines could induce modification of histones. The effect of ruxolitinib treatment was quantified with a screen of 21 histones. In the combined cell line analysis, H3K27me3 and H3K36me2 were significantly increased. For validation of histone methylation, three histone marks of primed and active transcription (H3K4me2, H3K4me3, and H3K27ac) were studied in detail with immunoprecipitation and sequencing. A clear differential was seen for H3K4me3 and H3K27ac as two marks of active transcription. Overall, results in cell lines showed that ruxolitinib had an immediate effect on the histone landscape with a significant increase in methylation and acetylation. However, the transcriptome of the cell showed a reduction in the expression of genes involved in cell signaling pathways. The epigenetic repriming of the cell was not immediately reflected in the transcriptome (60).

MAJIC: A randoMised study of best Available therapy versus JAK Inhibition in patients with high risk Polycythaemia Vera of Essential Thrombocythaemia who are resistant or intolerant to HydroxyCarbamide (ISRCTN61925716) evaluated the efficacy of ruxolitinib versus best available therapy in a second-line setting in PV and ET. With the MAJIC patient samples, the aim was to examine histone modification in MPN patients on ruxolitinib or BAT and to investigate for any association with clinical outcome. Paired samples from 51 patients in the trial were investigated. For some, all methylation histone marks increased with treatment, for others, all decreased, while in others, there were increases and decreases.

There was no change in any histone mark associated with a treatment arm in either disease group. However, when modifications within the same lysine were examined, methylation marks were decreased in follow-up samples compared to trial entry. The decrease in H3K36 marks was significant in ruxolitinib patients but not BAT patients, whereas the decrease in H3K4 marks was significant in the BAT patients but not the ruxolitinib treated. However, when looking at histone marks in isolation, at baseline and follow-up, high levels at trial entry of H3K4m2 and H3K4me3 were associated with lack of response to ruxolitinib. This study reflects the heterogeneous patient population, and the evolving histone landscape during prolonged therapy suggests a dynamic process of transcriptional control reflective of the role of therapy to modify transcription and suppress proliferation (60).

## Further study

Further investigation of the epigenome in MPNs is certainly warranted to understand the diseases and the factors that are involved in progression to acute leukemia and in the case of PV and ET to MF. A recent study explored the role of high-mobility group A1 (HMGA1), a chromatin regulator, in MPN disease progression in human samples and mouse models. HMGA1 is upregulated in MPN with highest levels after transformation. In J*AK2*V617F mouse models, loss of a single Hmga1 allele prevents progression to MF and HMGA1 depletion enhances responses to ruxolitinib preventing MF and increasing survival in the mice (61). This is of further interest in elucidating the pathogenesis of MPNs.

## Epigenetic therapies

As there are differences in the epigenome in MPNs and further alterations associated with progression, therapeutic modulation of epigenetically deregulated pathways may present an opportunity for targeted therapy in MPN patients at various stages of their disease pathway.

**Figure 3:**
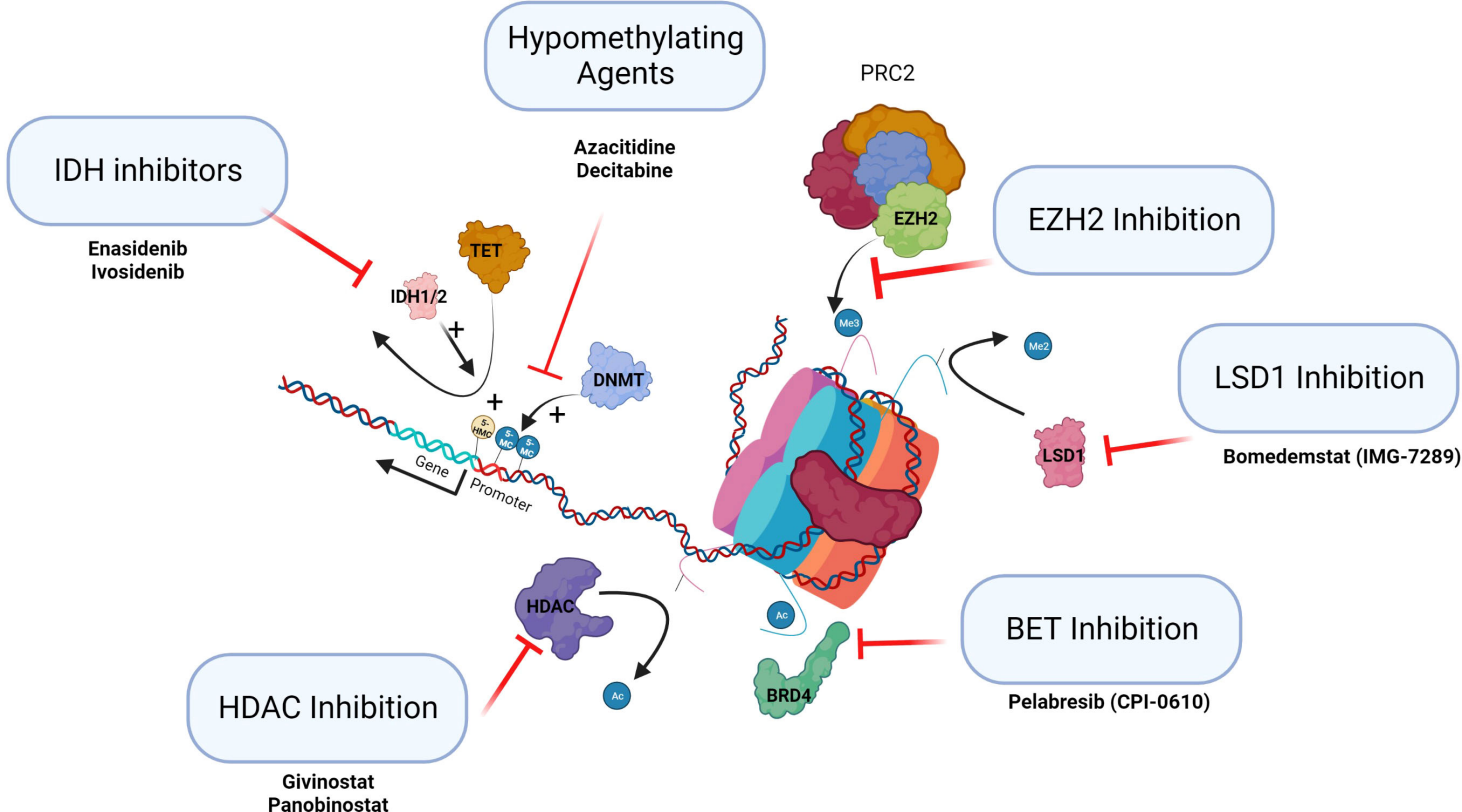
Epigenetic therapies in clinical trials in MPN. This figure summarizes the classes of epigenetic drugs being considered for or recently in trial for MPN patients.

There are a number of agents in use or in development that have or may have potential in the future in treating MPNs. Hypomethylating agents such as azacytidine, a cytosine nucleotide analog, is incorporated into RNA inhibiting metabolism and protein synthesis (see hypomethylating agents, **Figure 3**). However, it has demethylation activity (62). It is widely used in the management of myelodysplastic syndromes and AML. In the context of treatment of progression of MPN to AML, it has some utility currently.

Decitabine, another hypomethylating agent, a deoxycytidine analog, which is incorporated into DNA resulting in binding of methyltransferase and inactivation. However, at lower doses, it has a hypomethylating activity and reactivates silenced genes. It is therefore a useful agent with this function in the treatment of myeloid malignancies (63). In *JAK*2V617F-positive cell lines treated with decitabine, H3Y41ph levels were lowered and H3K9me2 levels increased at the NFE2 locus, normalizing NFE2 expression (46). These hypomethylating agents may therefore have further potential in treatment of MPNs.

Specific inhibitors of the epigenetic regulators *IDH*1/2 and *EZH2* are becoming available. Ivosidenib and enasidenib, IDH1 and 2 inhibitors, have been approved and licensed for AML (64). Tazemetostat is the first EZH2 inhibitor approved by the FDA and targets both wild-type and mutant EZH2 inducing cell cycle arrest. The use of EZH*2* inhibitors to date has been trialed in lymphoid malignancy where mutations are gain of function (65). It will be important to establish if inhibition of EZH2 may potentially drive selection of antecedent myeloid clones to establish MPN, MDS, or AML (see **Figure 3**, IDH inhibitors and EZH inhibition). The use of these targeted agents in MPN as inhibitors of the epigenetic regulators that are frequently mutated needs careful assessment.

There are a number of HDACis in clinical use (HDAC inhibition, **Figure 3**). Vorinostat has been trialed in PV and ET with some response, but the doses used in this trial were probably too high (53). Panobinostat, a pan HDACi, is used in the treatment of multiple myeloma, and romidepsin, a class 1 HDACi, is used as monotherapy in cutaneous T-cell lymphoma and in peripheral T-cell lymphoma. Toxicity of HDACi has been a consistent issue in clinical studies. Givinostat may be better tolerated than other HDACis and appears to be efficacious in MPN. PV patients treated with givinostat in phase I/II studies demonstrated clinical benefit in approximately two-thirds of patients initially. Longer-term follow-up of patients with PV who had an initial response to the drug showed a consistent overall response rate of greater than 80% with a 4-year mean follow-up (66). These agents may have a potential future role in the treatment of MPNs by altering the epigenome.

Bromodomain and extra terminal (BET) protein family consists of multiple epigenetic reader proteins that regulate gene transcription through binding of acetylated histones resulting in the control of cellular processes, such as transcription and chromatin remodeling (BET inhibition, **Figure 3**) **(** 67). In mouse models of MF, BET inhibitors demonstrated responses resulting from the attenuation of NF-κB signaling. These agents alone or in combination with ruxolitinib are now completing clinical trials in MF.

The demethylation of histone H3 lysine 4 and lysine 9 mono and di methylation (H3K4me1/2 and H3K9me1/2) is specifically catalyzed by lysine-specific demethylase 1 (LSD1). Normal LSD1 function is critical for normal differentiation during hematopoiesis (LSD1 inhibition, **Figure 3**). In mouse models of MPN, inhibition of LSD1 showed prolonged survival and was selective in targeting the disease clone with improvements in hematological parameters (68). These agents are now in clinical trials.

## Conclusion

Reversible epigenetic changes and epigenetic regulation are part of the pathogenesis of MPNs both in the disease development and in progression. Epigenetic changes in MPN have been investigated with a variety of results. Effects of aging and treatment on the epigenome are revealing. However, there is room for much greater investigation and understanding of epigenetic changes and events involved in progression. Investigation of the epigenome shows that there are a variety of therapeutic pathways available and that can be explored further leading to targeted therapy.

## Author contributions

Both author conceived the concept, wrote the article and checked final version GG designed the figures. All authors contributed to the article and approved the submitted version.
